# Leishmaniases in the European Union and Neighboring Countries

**DOI:** 10.3201/eid2706.210239

**Published:** 2021-06

**Authors:** Eduardo Berriatua, Carla Maia, Cláudia Conceição, Yusuf Özbel, Seray Töz, Gad Baneth, Pedro Pérez-Cutillas, Maria Ortuño, Clara Muñoz, Zarima Jumakanova, Andre Pereira, Rafael Rocha, Begoña Monge-Maillo, Elkhan Gasimov, Yves Van der Stede, Gregorio Torres, Céline M. Gossner

**Affiliations:** Universidad de Murcia, Murcia, Spain (E. Berriatua, P. Pérez-Cutillas, M. Ortuño, C. Muñoz, Z. Jumakanova);; Universidade NOVA de Lisboa, Lisbon, Portugal (C. Maia, C. Conceição, A. Pereira, R. Rocha);; Ege University, Izmir, Turkey (Y. Özbel, S. Töz);; The Hebrew University of Jerusalem, Rehovot, Israel (G. Baneth);; Instituto Ramón y Cajal de Investigación Sanitaria, Madrid, Spain (B. Monge-Maillo);; World Health Organization Regional Office for Europe, Copenhagen, Denmark. (E. Gasimov);; European Food Safety Authority, Parma, Italy (Y. Van der Stede);; World Organisation for Animal Health, Paris, France (G. Torres);; European Centre for Disease Prevention and Control, Stockholm, Sweden (C.M. Gossner)

**Keywords:** *Leishmania*, leishmaniosis, leishmaniases, neglected diseases, parasites, zoonoses, emergence, surveillance, prevention, sand flies, vector-borne infections, European Union

## Abstract

A questionnaire survey of animal and human health authorities in Europe revealed that leishmaniases are not notifiable in all countries with autochthonous cases. Few countries implement surveillance and control targeting both animal and human infections. Leishmaniases are considered emergent diseases in most countries, and lack of resources is a challenge for control.

Leishmaniases are endemic in humans and animals in part of the European Union (EU) and its neighboring countries. *Leishmania* species in this region are *L. major*, *L. tropica,* and the *L. donovani* complex species (including *L. infantum* and *L. donovani* sensu stricto). All cause cutaneous leishmaniasis (CL); visceral leishmaniasis (VL) is caused mainly by *L. donovani* complex species. There is evidence that the risk for leishmaniases is increasing in some EU and neighboring countries (*1*). We conducted a questionnaire survey to gather information on the epidemiologic situation, surveillance, prevention and control measures, and drivers of emergence of animal and human leishmaniases in this region during 2010–2020.

## The Study

**Figure 1:**
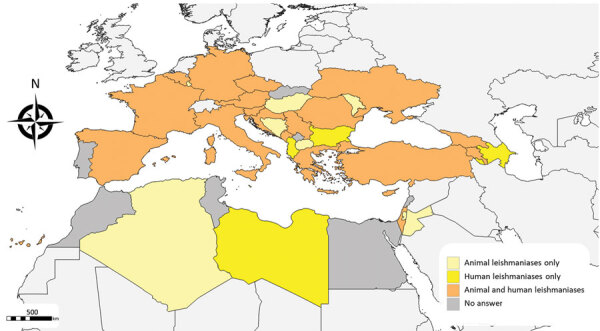
Geographic distribution of countries that responded to survey questionnaires about animal and human leishmaniases in Europe, 2020.

The survey included an animal leishmaniasis (AniL) questionnaire referring to *L. infantum* infections in domestic or wildlife hosts and a human leishmaniases (HumL) questionnaire referring to infections by *L. infantum*, *L. major*, *L. tropica* and *L. donovani* s.s. (Appendix). The target audience was the national focal points (national institutes or ministries) of the European Centre for Disease Prevention and Control, the World Health Organization, the European Food Safety Authority, and the World Organisation for Animal Health in countries in which leishmaniases are endemic or those with confirmed or suspected presence of sand fly vectors (*2*). These countries were Albania, Algeria, Armenia, Austria, Azerbaijan, Belgium, Bosnia and Herzegovina, Bulgaria, Croatia, Cyprus, Czechia, Egypt, France, Georgia, Germany, Greece, Hungary, Israel, Italy, Jordan, Kosovo, Lebanon, Libya, Liechtenstein, Luxembourg, Malta, Moldova, Montenegro, Morocco, North Macedonia, Palestine, Portugal, Romania, Serbia, Slovakia, Slovenia, Spain, Tunisia, Turkey, and Ukraine (Figure 1). The questionnaires were administered electronically using the EU survey tool and shared on September 11, 2020 (*3*). Twenty-seven countries (70%) replied to the AniL questionnaire and 24 countries (60%) to the HumL questionnaires; 19 countries (48%) replied to both (Table 1).

**Table 1:** Declared country status of leishmaniases surveillance and control, 2010–2020*

Country	Autochthonous			Notifiable			Surveillance			Control	
	Animal	Human		Animal	Human		Animal	Human		Animal	Human
Albania	NR	VL, CL		NR	Yes		NR	Yes		NR	No
Algeria	Yes	NR		Yes	NR		Yes	NR		Yes	NR
Armenia	Yes	VL		Yes	Yes		Yes	Yes		Yes	Yes
Austria	Not known	No		No	No		No	No		No	No
Azerbaijan	NR	VL, CL		NR	Yes		NR	Yes		NR	Yes
Belgium	No	No		No	No		No	Yes		No	No
Bosnia and Herzegovina	No	NR		Yes	NR		No	NR		No	NR
Bulgaria	NR	VL		NR	Yes		NR	Yes		NR	Yes
Croatia	Yes	VL, CL		Yes	Yes		No	Yes		No	Not known
Cyprus	Yes	VL, CL		Yes	Yes		Yes	Yes		No	No
Czechia	Not known	No		Yes	Yes		No	No		No	No
France	Yes	VL, CL		No	No		No	Yes		No	No
Georgia	Yes	VL		Yes	Yes		No	Yes		No	Yes
Germany	Not known	No		No	No		No	No		No	No
Greece	Yes	VL, CL		Yes	Yes		No	Yes		Yes	Yes
Hungary	Not known	NR		No	NR		No	NR		No	NR
Israel	Yes	VL, CL		Yes	Yes		No	Yes		No	Yes
Italy	Yes	VL, CL		Yes	Yes		Yes	Yes		Yes	Yes
Jordan	Yes	NR		Yes	NR		No	NR		No	NR
Libya	NR	VL, CL		NR	Yes		NR	Yes		NR	Yes
Luxemburg	Not known	NR		No	NR		No	NR		No	NR
Malta	NR	VL, CL		NR	Yes		NR	Yes		NR	Yes
Moldova	No	NR		Yes	NR		No	NR		No	NR
Montenegro	No	VL		Yes	Yes		No	Yes		Yes	No
North Macedonia	Yes	NR		Yes	NR		Yes	NR		Yes	NR
Palestine	Yes	NR		No	NR		No	NR		Yes	NR
Romania	Yes	No		No	Yes		No	No		No	Not known
Serbia	Yes	VL, CL		No	No		No	No		No	Yes
Slovenia	Yes	No		Yes	Yes		No	Yes		No	No
Spain	Yes	VL, CL		Regionally	Yes		Yes	Yes		No	Yes
Turkey	Yes	VL, CL		No	Yes		No	Yes		No	Yes
Ukraine	Yes	VL		Regionally	Yes		Yes	Yes		No	No

*Data source: questionnaires survey on animal and human leishmaniases to national focal points of the European Centre for Disease Prevention and Control, the World Health Organization, the European Food Safety Authority, and the World Organisation for Animal Health; survey conducted in 2020. CL, cutaneous leishmaniases; NR, no response; VL, visceral leishmaniases.

We reviewed the countries’ epidemiologic status with regards to autochthonous *Leishmania* spp. infections in animals and humans and clinical forms in humans. The mapping of the countries with autochthonous transmission matches previous published information with few discrepancies. For instance, according to the questionnaire, Bosnia and Herzegovina and Hungary do not have autochthonous canine leishmaniasis cases, although such cases have been described (*4*,*5*). Human cases of leishmaniasis due to *L. tropica* were reported in Cyprus and Serbia and due to *L. major* in Georgia; however, none of the literature presents concurring evidence (Table 2).

**Table 2:** Declared status of endemicity of *Leishmania* spp. affecting humans, by country

Country	*Leishmania *species			
	*L. infantum*	*L. major*	*L. tropica*	*L. donovani*
Albania	Yes	No	No	No
Armenia	Yes	No	No	No
Austria	No	No	No	No
Azerbaijan	Yes	Yes	Yes	No
Belgium	No	No	No	No
Bulgaria	Yes	No	No	No
Croatia	Not known	Not known	Not known	Not known
Cyprus	No	No	Yes	Yes
Czechia	No	No	No	No
France	Yes	No	No	No
Georgia	Yes	Yes	No	No
Germany	No	No	No	No
Greece	Yes	No	No	No
Israel	Yes	Yes	Yes	No
Italy	Yes	No	No	No
Libya	Yes	Yes	Yes	Not known
Malta	Yes	No	No	No
Montenegro	Yes	Not known	Not known	No
Romania	No	No	No	No
Serbia	Yes	Not known	Yes	No
Slovenia	No	No	No	No
Spain	Yes	No	No	No
Turkey	Not known	Not known	Yes	Not known
Ukraine	No	No	No	No

**Figure 2:**
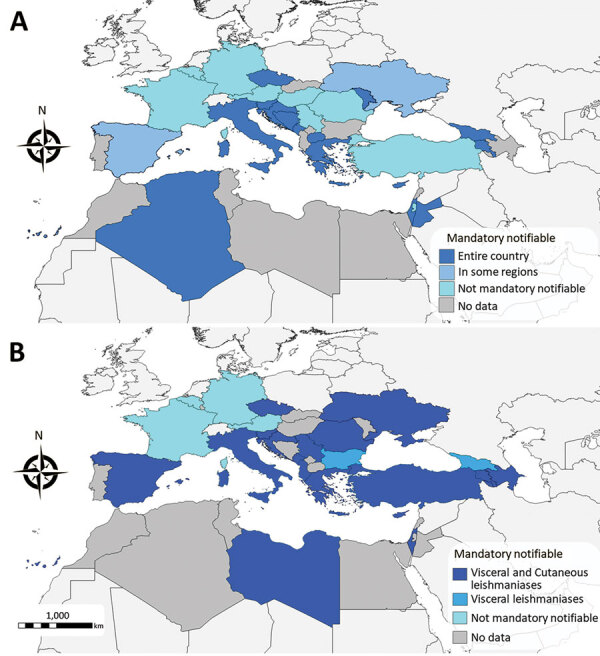
Geographic distribution of mandatory notification status for animal (A) and human (B) leishmaniases, 2020.

Animal leishmaniases are notifiable in 17 countries and human leishmaniases in 20 countries (Table 1; Figure 2). In Palestine and Turkey, AniL is not notifiable despite a high prevalence among dogs (*6*,*7*). Similarly, in France, neither AniL nor HumL are notifiable although the diseases are endemic in the south (*8*). Leishmaniases surveillance is not mandatory at the EU level which constitutes a limitation for successful control.

Seven countries conduct AniL surveillance (Table 1), indicative of its low priority among the animal health authorities. The target animal population for surveillance included symptomatic and asymptomatic dogs in Armenia, Cyprus, Italy, Spain, and Ukraine; we also studied wildlife in leishmaniasis foci in Spain. Testing subclinically infected dogs indicated awareness of their role as reservoirs of the parasite (*1*). Similarly, wild lagomorphs were the main reservoir of *L. infantum* in a HumL outbreak in Madrid in Spain (*9*). Surveillance of HumL is conducted in 19 countries, including all of those with autochthonous infections except Serbia (Table 1).

Antibody tests, including the immunofluorescence antibody test, ELISA, and the rapid immunochromatography test, are the main surveillance diagnostic methods used, followed by PCR. Antibody tests play a fundamental role in disease surveillance because they are relatively cheap and easy to use (*10*). However, their sensitivity to detect subclinical infections is lower than that of PCR tests (*10*), and they do not discriminate naturally infected from vaccinated dogs (*11*). PCR tests are ideal for epidemiologic studies to estimate *Leishmania* spp. infection prevalence in healthy hosts, but their diagnostic validity depends on the sample used, the DNA sequence target, and the PCR protocol. Standardization of PCR tests in leishmaniasis diagnosis is needed (*12*).

Of the 7 countries that have ongoing AniL prevention and control programs (Table 1), 5 use topical insecticides for dogs, 5 are diagnosing and treating leishmaniases in dogs, and 2 use canine leishmaniosis vaccines. In all countries, infected dogs may be euthanized on welfare grounds. Lack of funds and treatment costs were considered the most important AniL control challenges. Human leishmaniasis prevention and control activities are implemented in 12 countries (Table 1); for *L. infantum*, actions focused on the use of insecticides on dogs, and for *L. major*, *L. tropica*, and *L. donovani*, the common activity was the use of peridomiciliary and intradomiciliary insecticides. Lack of funds and capacity constraints are considered the main challenges for HumL. 

Although zoonotic *L. infantum* strategies are centered on preventing and eliminating infections in dogs, the main parasite reservoir host, we found that insecticides and treatments are not fully effective and are expensive, and so provided to a relatively small proportion of dogs. Leishmaniasis control needs the One Health approach to account for the complexity of its transmission cycle involving humans, domestic animals, wildlife, and sand fly vectors (*13*).

Animal leishmaniases are considered emergent diseases in Cyprus and Jordan and in parts of Algeria, Armenia, France, Georgia, Jordan, Montenegro, North Macedonia, Romania, Slovenia, Turkey, and Ukraine. The most important AniL emergence risk factor is the lack of control. Human leishmaniases are considered emerging diseases in Cyprus, Libya and Malta and in parts of Albania, Austria, Armenia, Azerbaijan, Georgia, Israel, Italy, Montenegro, and Spain. The main risk factors for HumL emergence are vector expansion for *L. infantum*, and movement of infected persons between countries for *L. major*, *L. tropica*, and *L. donovani*.

In general, the perceived increasing risk for AniL and HumL was in line with the literature. In the EU and its neighborhood, the risks include movement of humans and dogs, increased number of immunosuppressed patients, climate warming, and other environmental changes affecting vector and reservoir host distribution (*1*,*14*). Limitations associated with existing surveillance and control programs, along with the fact that leishmaniases are often regarded as a local problem rather than a transnational problem, are deemed major obstacles to overcome to prevent leishmaniases emergence in the EU and its neighborhood.

## Conclusions

Leishmaniases are considered widespread, endemic, or emerging infections in the EU and its neighborhood, yet are neglected and underreported because they are low priority at the country and EU level. Our study revealed a clear need to strengthen leishmaniasis prevention and control programs in the EU and its neighborhood. We recommend analysis of leishmaniasis incidence in the region for an objective assessment of disease emergence, and also improvement of prevention and control programs based on a robust surveillance and following a One Health approach.

Appendix Questionnaires used in a study of animal leishmaniases in 2010–2020 in the European Union and neighboring countries.
