# Supplementary material for: Leishmaniases in the European Union and Neighboring Countries
Source: Emerg Infect Dis. 2021 Jun;27(6):1723–7. doi: 10.3201/eid2706.210239 (PMC8153892; doi:10.3201/eid2706.210239)
Supplement: Appendix — Questionnaires used in a study of animal leishmaniases in 2010–2020 in the European Union and neighboring countries. [file 21-0239-Techapp-s1.pdf]

Article DOI: https://doi.org/10.3201/eid2706.210239

Leishmaniases in the European Union and Neighboring Countries

Appendix

Shown on the following pages are 2 surveys used to gather information on the epidemiologic situation, surveillance, prevention and control measures, and drivers of emergence of animal and human leishmaniases in Europe during 2010–2020. The first is an animal leishmaniasis questionnaire referring to *Leishmania infantum* infections in domestic or wildlife hosts. The second is a human leishmaniases questionnaire referring to infections by *L. infantum*, *L. major*, *L. tropica*, and *L. donovani* sensu stricto.

ANIMAL LEISHMANIASIS BY LEISHMANIA INFANTUM IN THE EUROPEAN UNION AND ITS NEIGHBOURHOOD

Fields marked with * are mandatory.

A - INTRODUCTION

Dear Participants,

The European Centre for Disease Prevention and Control (ECDC), in collaboration with the European Food Safety Authority (EFSA), the World Health Organization Regional Office for Europe (WHO EURO) and the World Organisation for Animal Health (OIE), initiated the review of the epidemiological situation of human and animal leishmaniasis in the European Union and its neighbourhood.

To collect data on the surveillance, control, diagnosis and treatment of leishmaniasis, we would like you to complete the following questionnaire by 30 September. This will take you approximately 15 minutes. Note that this questionnaire targets the public health authorities and focuses on human leishmaniasis. A complementary questionnaire focusing on animal leishmaniasis will be sent to the animal health authorities in your country.

Based on the responses to this questionnaire and a literature review, a technical report will be prepared. If you want to receive the finalised technical report and have your contribution acknowledged in the technical report, please express this in the first part of the questionnaire.

B - ECDC DATA PRIVACY STATEMENT

* Do you agree with the following ECDC privacy statement? *By agreeing with ECDC privacy statement and answering this questionnaire, you agree that your answers are used in the technical report that will be produced. Note that ECDC may contact you afterwards for clarification.*

- ◯ Yes

Download

ECDC_Data_Privacy_Statement.pdf

You can only proceed with the questionnaire by accepting ECDC data privacy statement.

C - GENERAL INFORMATION

Name (only visible to ECDC). This information will be treated as strictly confidential.

Email (only visible to ECDC). This information will be treated as strictly confidential.

Affiliation

* Country

- Albania
- Algeria
- Andorra
- Armenia
- Austria
- Azerbaijan
- Belgium
- Bosnia and Herzegovina
- Bulgaria
- Croatia
- Cyprus
- Czechia
- Denmark
- Egypt
- Estonia
- Finland
- France
- Georgia
- Germany
- Greece
- Hungary
- Ireland
- Israel
- Italy
- Jordan
- Kosovo
- Latvia
- Lebanon
- Libya
- Liechtenstein
- Lithuania

- Luxembourg
- Macedonia
- Malta
- Moldova
- Monaco
- Montenegro
- Morocco
- Netherlands
- North Macedonia
- Palestine
- Poland
- Portugal
- Romania
- Serbia
- Slovak Republic
- Slovenia
- Spain
- Sweden
- Switzerland
- Syria
- Tunisia
- Turkey
- Ukraine

* Do you want to receive a final copy of the technical report (expected early 2021)?

- Yes
- No

* Do you want to have your contribution acknowledged in the technical report that will be prepared?
If yes, please make sure you provide your name and affiliation.

- Yes
- No

D - SURVEILLANCE OF ANIMAL LEISHMANIASIS

* 1. Have **autochthonous cases of animal** (dog or other domestic or wildlife) **leishmaniasis** been identified in your country **since 2010**?
*Animal leishmaniasis in this context refers to a laboratory confirmed infection case by **Leishmania infantum**. Other domestic animals include cats, horses, etc. Wildlife include foxes, wolves, rodents, rabbits, hares, etc. Autochthonous cases result from existing natural leishmania transmission in the country.*

- Yes
- No
- I don't know

Selecting "No" or "I don't know" skips question 2

2. Which of the following **clinical presentations** of autochthonous leishmaniasis have been **identified** in animals in your country? You may choose more than one.

- ☐ Visceral
- ☐ Cutaneous
- ☐ I don't know

* 3. Is **animal** (canine or other) leishmaniasis a **mandatory notifiable disease** in your country?
A notifiable disease is required by law to be reported to government authorities.

- ○ Yes, in the entire country
- ○ Yes, in some regions only
- ○ No
- ○ I don't know

Please specify which region(s)

Selecting "No" or "I don't know" skips question 4

4. In which **host species** is leishmaniasis **notification** mandatory?

- ○ All species (dogs and other domestic animals and wildlife)
- ○ Dogs and other domestic animals only
- ○ Dogs only
- ○ I don't know

5. Is **SURVEILLANCE** of **animal leishmaniasis** implemented in your country?
Surveillance refers to the systematic and continuous collection, management, analysis, interpretation and reporting of infection/disease data to drive health actions.

- ○ Yes
- ○ No
- ○ I don't know

Selecting "No" or "I don't know" skips questions from 6 to 11

6. What **type of SURVEILLANCE** system of animal leishmaniasis is implemented in your country?

- ○ Comprehensive: by all providers of veterinary care, official and private in a particular geographical area
- ○ Sentinel: by only a subset of veterinary care providers should report cases
- ○ I don't know

7. What are the **SURVEILLANCE data providers** for animal leishmaniasis in your country? You may choose more than one.

- ☐ Laboratories from the official veterinary services or laboratories accredited by the National Veterinary Authority
- ☐ Laboratories from specialised, private companies not acredited by the National Veterinary Authority
- ☐ Veterinary practices and veterinary hospitals

- [ ] Other
- [ ] I don't know

Please specify:

8. What **type of SURVEILLANCE data** from animal leishmaniasis cases is reported in your country? You may choose more than one.
Please note that the question also applies to non-endemic regions/countries in the event that a case was diagnosed.

- [ ] Clinical
- [ ] Epidemiological
- [ ] Laboratorial
- [ ] Other
- [ ] I don't know

Please specify:

9. In addition to surveillance of clinical cases of leishmaniasis, is **SURVEILLANCE OF ASYMPTOMATIC (subclinical) INFECTIONS** implemented in your country?

- ( ) Yes
- ( ) No
- ( ) I don't know

Selecting "No" or "I don't know" skips question 9

10. What is the **target animal population** on which **SURVEILLANCE** of infection/disease is done? You may select more than one.

- [ ] Privately owned dogs (pets, farm, hunting)
- [ ] Animal shelter and kennel dogs not privately owned (e.g. police dogs)
- [ ] Captured strays and rescued dogs for rehoming
- [ ] Wild canids (e.g. foxes)
- [ ] Wild lagomorphs (e.g. rabbits)
- [ ] Wild rodents
- [ ] Other
- [ ] I don't know

Please specify:

11. What are the **diagnostic techniques used for SURVEILLANCE**? You may select more than one.

- Serology (antibody detection) by IFAT (indirect immunofluorescence test)
- Serology by ELISA (Enzyme linked immunosorbent assay)
- Serology by DAT (direct agglutination test)
- Serology by rapid immunochromatography (e.g. IDEXX snap test)
- PCR (Polymerase chain reaction) in blood samples
- PCR of skin/tissue samples
- PCR of conjunctival/oral swabs
- Microscopy of biological samples with or without prior culture
- Necropsy and PCR of biological samples
- Other
- I don't know

Please specify:

E - PREVENTION AND CONTROL OF ANIMAL LEISHMANIASIS

12. Following surveillance results, is there a **PREVENTION AND CONTROL PROGRAMME** implemented in your country by **veterinary authorities** against animal leishmaniasis?
Prevention and control actions may include: treatment of infected animals, canine vaccination, culling of infected animals, testing animals coming from endemic areas, use of insecticides and mechanical barriers against sand fly vectors.

- Yes
- No
- I don't know

Selecting "No" or "I don't know" skips questions 13 and 14

Which of the following actions are taken? You may select more than one.

	Yes	No	I don´t know
Treatment of infected dogs			
Vaccination of dogs			
Insecticide use in dogs			
Diagnostic testing of pet dogs			
Diagnostic testing of pet dogs travelling from endemic areas (e.g. after holidays)			
Collect and test stray/abandoned dogs			
Collect and test wildlife (foxes, rabbits, etc.)			
Euthanasia of dogs and other infected animals			

Other	◯	◯	◯

Please specify:

13. Do you have national **PREVENTION AND CONTROL official guidelines** for animal leishmaniasis?

- ◯ Yes
- ◯ No
- ◯ I don't know

Please provide the link where to find these guidelines:

14. Are you aware of **intersectorial ("One Health") collaboration** between animal, human and environmental sectors to **PREVENT AND CONTROL** leishmaniasis in your country?

- ◯ Yes
- ◯ No
- ◯ I don't know

Please describe:

15. What are in your opinion the **challenges for the PREVENTION AND CONTROL** of leishmaniasis in animals in your country? Please rate from 0 (not important) to 3 (very important).

	0	1	2	3	I don't know
Lack of legislation from responsible authorities	◯	◯	◯	◯	◯
Lack of political willingness/awareness	◯	◯	◯	◯	◯
Lack of funding	◯	◯	◯	◯	◯
Collaborative constraints (between stakeholders)	◯	◯	◯	◯	◯
Operational capacity constraints (Knowledge, people, equipment)	◯	◯	◯	◯	◯
Lack of national/international regulation against culling infected/sick dogs/animals	◯	◯	◯	◯	◯
Limited availability of diagnostic techniques	◯	◯	◯	◯	◯

High cost of diagnosis	○	○	○	○	○
Limited availability of treatments	○	○	○	○	○
High cost of treatment	○	○	○	○	○
Limited availability of vaccines	○	○	○	○	○
Limited availability of insecticides	○	○	○	○	○
No regulatory basis for insecticide use	○	○	○	○	○
Other	○	○	○	○	○

Please specify:

F - DIAGNOSIS AND TREATMENT OF ANIMAL LEISHMANIASIS

16. Are there any **official guidelines** in your country for animal leishmaniasis **DIAGNOSIS**?

- ○ Yes
- ○ No
- ○ I don't know

Please provide the link where to find these guidelines:

17. Which of the following **LABORATORY TECHNIQUES** are employed in your country for animal leishmaniasis **DIAGNOSIS** by governmental or private laboratories?
Please note that this does not necessarily imply that animal leishmaniosis surveillance is being carried out in your country.

	Yes	No	I don´t know
Serology (antibody detection) by IFAT(indirect immunofluorescence antibody test)	○	○	○
Serology by ELISA (enzyme-linked immunosorbent assay)	○	○	○
Serology by DAT (direct agglutination test)	○	○	○
Serology by rapid immunochromatography (e.g. IDEXX snap test)	○	○	○
PCR (Polymerase chain reaction) of blood samples	○	○	○

PCR of skin/tissue samples	○	○	○
Microscopy of biological samples with or without prior culture	○	○	○
Other	○	○	○

Please specify:

18. Are there any **guidelines** in your country for animal leishmaniasis **TREATMENT**?

- Yes
- No
- I don't know

Please provide the link where to find these guidelines:

19. What are the **DRUGS** (medicines) used for **animal leishmaniasis treatment** in your country?

	Yes	No	I don´t know
Meglumine antimoniate (E.g. Glucantime®)	○	○	○
Allopurinol	○	○	○
Miltefosine	○	○	○
Sodium stibogluconate (E.g. Pentostam®)	○	○	○
Aminosidine	○	○	○
Immunomodulatory drugs (e.g. Domperidone)	○	○	○
Other	○	○	○

Please specify

G - DRIVERS OF EMERGENCE OF ANIMAL LEISHMANIASIS

Emergence refers to the establishment of infection/disease in a previously free area or to an increase in incidence of infection/disease in an endemic area. Disease emergence is also considered when the rise in incidence occurs as a result of improved and wider diagnosis.

20. Is **animal leishmaniasis emerging** in your country?

- Yes, in all the country
- Yes, in some regions
- No
- I don't know

Please specify which region(s)

21. **WHICH** of the following **DRIVERS** do you think that are important in your country? Please rate from 0 (not important) to 3 (very important).

	0	1	2	3
Pet animals travelling to Leishmania endemic areas				
Importation of pet animals from Leishmania endemic areas				
Immigration of Leishmania infected people				
Sand fly vector expansion into previously-free areas as a result of climate change				
Environmental changes other than climate change, with an impact in vectors and reservoir hosts (e. g. urbanization)				
Insufficient/lack of surveillance at regional/country level				
Insufficient/lack of prevention and control at regional/country level				
Other				

Please comment:

H - CONCLUDING REMARKS

Please provide any additional information you consider relevant:

HUMAN LEISHMANIASIS IN THE EUROPEAN UNION AND ITS NEIGHBOURHOOD

Fields marked with * are mandatory.

A - INTRODUCTION

Dear Participants,

The European Centre for Disease Prevention and Control (ECDC), in collaboration with the European Food Safety Authority (EFSA), the World Health Organization Regional Office for Europe (WHO EURO) and the World Organisation for Animal Health (OIE), initiated the review of the epidemiological situation of human and animal leishmaniasis in the European Union and its neighbourhood.

To collect data on the **surveillance, control, diagnosis and treatment of leishmaniasis, we would like you to complete the following questionnaire by 30 September.** This will take you approximately 15 minutes. Note that this questionnaire targets the public health authorities and focuses on human leishmaniasis. A complementary questionnaire focusing on animal leishmaniasis will be sent to the animal health authorities in your country.

Based on the responses to this questionnaire and a literature review, a technical report will be prepared. If you want to receive the finalised technical report and have your contribution acknowledged in the technical report, please express this in the first part of the questionnaire.

B - ECDC DATA PRIVACY STATEMENT

* Do you agree with the following ECDC privacy statement? *By agreeing with ECDC privacy statement and answering this questionnaire, you agree that your answers are used in the technical report that will be produced. Note that ECDC may contact you afterwards for clarification.*

- ◯ Yes

Download:

ECDC_Data_Privacy_Statement.pdf

You can only proceed with the questionnaire by accepting ECDC data privacy statement.

C - GENERAL INFORMATION

Name (only visible to ECDC). This information will be treated as strictly confidential

Email (only visible to ECDC). This information will be treated as strictly confidential

Affiliation

* Country

- Albania
- Algeria
- Andorra
- Armenia
- Austria
- Azerbaijan
- Belgium
- Bosnia and Herzegovina
- Bulgaria
- Croatia
- Cyprus
- Czechia
- Denmark
- Egypt
- Estonia
- Finland
- France
- Georgia
- Germany
- Greece
- Hungary
- Ireland
- Israel
- Italy
- Jordan
- Kosovo
- Latvia
- Lebanon
- Libya
- Liechtenstein
- Lithuania

- Luxembourg
- Macedonia
- Malta
- Moldova
- Monaco
- Montenegro
- Morocco
- Netherlands
- North Macedonia
- Palestine
- Poland
- Portugal
- Romania
- Serbia
- Slovak Republic
- Slovenia
- Spain
- Sweden
- Switzerland
- Syria
- Tunisia
- Turkey
- Ukraine

* Do you want to receive a final copy of the technical report (expected early 2021)?

- Yes
- No

* Do you want to have your contribution acknowledged in the technical report that will be prepared?

If yes, please make sure you provide your name and affiliation.

- Yes
- No

D - SURVEILLANCE OF HUMAN LEISHMANIASIS

* 1. Have **autochthonous cases** of human leishmaniasis been **identified in your country** since 2010?

A case of human leishmaniasis is a laboratory confirmed infection. Autochthonous cases result from existing natural Leishmania transmission in the country.

- Yes
- No
- I don't know

Selecting "No" or "I don't know" skips questions 2 and 3

2. Which of the following **clinical presentations** of **autochthonous leishmaniasis** have been **identified in your country**? You may choose more than one.

- ☐ Visceral
- ☐ Cutaneous
- ☐ Mucocutaneous*
- ☐ I don't know

3. Which of the following ***Leishmania*** **species** are considered **endemic among humans in all or some parts of your country**? You may choose more than one

3.1 ***Leishmania infantum*** (human and canine visceral and cutaneous leishmaniosis)

- ○ Yes
- ○ No
- ○ I don't know

3.2 ***Leishmania major*** (human cutaneous leishmaniosis)

- ○ Yes
- ○ No
- ○ I don't know

3.3 ***Leishmania tropica*** (human cutaneous leishmaniosis)

- ○ Yes
- ○ No
- ○ I don't know

3.4 ***Leishmania donovani*** (human visceral and cutaneous leishmaniosis)

- ○ Yes
- ○ No
- ○ I don't know

4. Is **human leishmaniasis** a **mandatory notifiable disease** in your country?
A notifiable disease is required by law to be reported to government authorities.

* 4.1 **Visceral**

- ○ Yes, in the entire country
- ○ Yes, in some regions only
- ○ No
- ○ I don't know

Please specify which region(s)

* 4.2 **Cutaneous**

- ○ Yes, in the entire country

** Note added after the completion of the questionnaire survey: by mistake the authors included mucocutaneous instead of mucosal leishmaniosis. The specific answers to mucocutaneous were therefore not included in the results presented in the manuscript "Surveillance, prevention and control of leishmaniases in the European Union and its neighborhood".*

- Yes, in some regions only
- No
- I don't know

Please specify which region(s)

* 4.3 **Mucocutaneous** *

- Yes, in the entire country
- Yes, in some regions only
- No
- I don't know

Please specify which region(s)

5. Is **SURVEILLANCE** of **human leishmaniasis** implemented in your country?
Surveillance refers to the systematic and continuous collection, management, analysis, interpretation and reporting of infection/disease data to drive health actions.

- Yes
- No
- I don't know

Selecting "No" or "I don't know" skips questions from 6 to 10

What **type of SURVEILLANCE** system of human leishmaniasis is implemented in your country?

- Comprehensive: All healthcare providers of at least one level of care are reporting their cases, e.g. all hospitals report cases
- Sentinel: Only a subset of healthcare providers report cases
- I don't know

6. What are the **SURVEILLANCE data providers** for human leishmaniasis in your country? You may choose more than one.

- Hospitals
- Local health care centers
- Private physicians
- Laboratories of the Public Health authority or acredited by the Public Health authority
- Laboratories from specialised, private companies not acredited by the Public Health authority
- Other
- I don't know

Please specify:

** Note added after the completion of the questionnaire survey: by mistake the authors included mucocutaneous instead of mucosal leishmaniosis. The specific answers to mucocutaneous were therefore not included in the results presented in the manuscript "Leishmaniases in the European Union and its neighbourhood: Neglected zoonotic diseases with increasing public health risk"*

7. What **type of SURVEILLANCE data** from human leishmaniasis cases is reported in your country? You may choose more than one.
Please note that the question also applies to non-endemic countries in the event that a case was diagnosed.

- ☐ Clinical
- ☐ Epidemiological
- ☐ Laboratorial
- ☐ Other
- ☐ I don't know

Please specify:

8. In addition to surveillance of clinical leishmaniasis, is **SURVEILLANCE OF ASYMPTOMATIC (subclinical)** infections implemented in your country?

- ○ Yes
- ○ No
- ○ I don't know

Selecting "No" or "I don't know" skips question 9

9. What is the **target human population** on which **SURVEILLANCE** of **ASYMPTOMATIC** leishmaniasis is done? You may select more than one.

- ☐ Blood donors
- ☐ Organ donors
- ☐ Other target risk groups: e.g. HIV+ patients, intravenous drug users, etc.
- ☐ Samples of patients admitted to hospital for reasons other than leishmaniosis
- ☐ People coming from endemic zones (travellers, migrants)
- ☐ Other
- ☐ I don't know

Please specify:

10. What are the **diagnostic techniques used for SURVEILLANCE of human leishmaniasis infection (clinical and subclinical)** in your country? You may select more than one.

- ☐ Serology (antibody detection) by IFAT (indirect immunofluorescence test)
- ☐ Serology by rapid immunochromatography (e.g. rK39 test)
- ☐ Serology by ELISA (Enzyme-linked immunosorbent assay)
- ☐ Serology by DAT (direct agglutination test)

- [ ] PCR (Polymerase chain reaction) of blood samples
- [ ] PCR of skin/tissue samples
- [ ] Microscopy of biological samples with or without prior culture
- [ ] Other
- [ ] I don't know

Please specify:

E - PREVENTION AND CONTROL OF HUMAN LEISHMANIASIS

11. Following surveillance results, is there a **PREVENTION AND CONTROL programme** implemented in your country **by national health authorities** against visceral and cutaneous leishmaniasis?
Prevention and control actions include for example: treatment of animal and human cases, use of insecticides and mechanical barriers against sand fly vectors by people or in the environment, canine vaccination, culling of animal reservoirs, testing people and animals coming from endemic areas (travellers and migrants).

- ◯ Yes
- ◯ No
- ◯ I don't know

Selecting "No" or "I don't know" skips questions 12 and 13

Which of the following **prevention and control actions** are taken? You may select more than one.

11.1. For ***L. infantum*** (human and canine visceral leishmaniasis).

	Yes	No	I don´t know
Treatment of infected people	◯	◯	◯
Treatment of infected dogs	◯	◯	◯
Vaccination of dogs	◯	◯	◯
Insecticide use in dogs	◯	◯	◯
Collect and test stray/abandoned dogs	◯	◯	◯
Collect and test wildlife (foxes, rabbits, etc.)	◯	◯	◯
Euthanasia of dogs and other infected animals	◯	◯	◯
Testing people and animals coming from endemic areas (travellers and migrants)	◯	◯	◯
Other	◯	◯	◯

Please specify:

11.2 For ***L. major*** (cutaneous leishmaniasis in humans).

	Yes	No	I don't know
Treatment of cases			
Leishmanisation: intradermal inoculation of live Leishmania to produce a self-healing lesion and stimulate immunity against reinfection			
Insecticide application in the peridomiciliary environment			
Mechanical barriers for vectors in the peridomiciliary environment			
Insecticide application in the intradomiciliary environment			
Use of insecticide impregnated bed nets			
Use of insecticide impregnated bed linen			
Destruction of animal reservoir habitat (e.g. rodent burrows)			
Testing people and animals coming from endemic areas (travellers and migrants)			
Other			

Please specify:

11.3 For ***L. tropica*** (cutaneous leishmaniasis in humans).

	Yes	No	I don't know
Treatment of cases			
Leishmanisation: intradermal inoculation of live Leishmania to produce a self-healing lesion and stimulate immunity against reinfection			
Insecticide application in the peridomiciliary environment			
Mechanical barriers for vectors in the peridomiciliary environment			
Insecticide application in the intradomiciliary environment			
Use of insecticide impregnated bed nets			
Use of insecticide impregnated bed linen			

Destruction of animal reservoir habitat (e.g. rodent burrows)	○	○	○
Testing people and animals coming from endemic areas (travellers and migrants)	○	○	○
Other	○	○	○

Please specify:

11.4 For ***L. donovani*** (visceral and cutaneous leishmaniasis in humans).

	Yes	No	I don't know
Treatment of cases	○	○	○
Leishmanisation: intradermal inoculation of live Leishmania to produce a self-healing lesion and stimulate immunity against reinfection	○	○	○
Insecticide application in the peridomiciliary environment	○	○	○
Mechanical barriers for vectors in the peridomiciliary environment	○	○	○
Insecticide application in the intradomiciliary environment	○	○	○
Use of insecticide impregnated bed nets	○	○	○
Use of insecticide impregnated bed linen	○	○	○
Destruction of animal reservoir habitat (e.g. rodent burrows)	○	○	○
Testing people and animals coming from endemic areas (travellers and migrants)	○	○	○
Other	○	○	○

Please specify:

12. Do you have national **PREVENTION AND CONTROL official guidelines** for human leishmaniasis?

- ○ Yes
- ○ No
- ○ I don't know

Please provide the link where to find these guidelines:

13. Are you aware of **intersectorial ("One Health") collaboration** between animal, human and environmental sectors to **PREVENT AND CONTROL** leishmaniasis in your country?

- Yes
- No
- I don't know

Please describe:

14. What are the **challenges** for the **PREVENTION AND CONTROL** of leishmaniasis in humans in your country? Please rate from 0 (not important) to 3 (very important).

	0	1	2	3	I don't know
Lack of legislation from responsible authorities					
Lack of political willingness/awareness					
Financial constraints					
Collaborative constraints (between stakeholders)					
Operational capacity constraints (knowledge, people, equipment)					
National/EU regulation against culling infected/sick dogs /animals					
Limited availability of diagnostic techniques					
High cost of diagnosis					
Limited availability of treatments					
High cost of treatment					
Limited availability of vaccines					
Limited availability of rodenticides					
No regulatory basis for rodenticide use					
Limited availability of insecticides					
No regulatory basis for insecticide use					
Environmental interventions to destroy the habitat of reservoirs					
Other					

Please specify:

F - DIAGNOSIS AND TREATMENT OF HUMAN LEISHMANIASIS

15. Are there any **official guidelines** in your country for human leishmaniasis **DIAGNOSIS**?

- Yes
- No
- I don't know

Please provide the link where to find these guidelines:

16. Which of the following **LABORATORY TECHNIQUES** are employed in your country for human leishmaniasis **DIAGNOSIS** by governmental or private laboratories?
Please note that this does not necessarily imply that human leishmaniosis surveillance is being carried out in your country.

	Yes	No	I don't know
Serology (antibody detection) by IFAT(indirect immunofluorescence antibody test)			
Serology by ELISA (enzyme-linked immunosorbent assay)			
Serology by DAT (direct agglutination test)			
Serology by rapid immunochromatography (e.g. rk39 test)			
PCR (Polymerase chain reaction) in blood samples			
PCR in skin/tissue samples			
Microscopy of biological samples with or without prior culture			
Other			

Please specify:

17. Are there any **national guidelines** in your country for human leishmaniasis **TREATMENT**?

- Yes
- No

- I don't know

Please provide the link where to find these guidelines:

18. What are **the DRUGS (medicines)** used for **human leishmaniasis treatment** in your country?

	Yes	No	I don't know
Liposomal amphotericin B			
Amphotericin B deoxicholate			
Sodium stibogluconate (E.g. Pentostam®)			
Meglumine antimoniate (E.g. Glucantime®)			
Miltefosine			
Other			

Please specify

G - DRIVERS OF EMERGENCE OF HUMAN LEISHMANIASIS

Emergence refers to the establishment of infection/disease in a previously free area or to an increase in incidence of infection/disease in an endemic area. Disease emergence is also considered when the rise in incidence occurs as a result of improved and wider diagnosis.

19. Is **cutaneous and/or visceral leishmaniasis emerging** in your country?

- Yes, in all the country
- Yes, in some regions
- No
- I don't know

Please specify which region(s)

Selecting "No" or "I don't know" skips question 20

20. **WHICH** of the following **DRIVERS** do you think that are important in your country? Please rate from 0 (not important) to 3 (very important) for the *Leishmania* species endemic in your country.

20.1 ***L. infantum*** (human and canine visceral and cutaneous leishmaniasis)

	0	1	2	3
Infected people and animals coming from endemic areas (travellers and migrants)	○	○	○	○
Sand fly vector expansion into previously-free areas as a result of climate change	○	○	○	○
Environmental changes other than climate change, with an impact in vectors and reservoir hosts (e. g. urbanization, agricultural projects)	○	○	○	○
Insufficient/lack of surveillance at regional/country level	○	○	○	○
Insufficient/lack of prevention and control at regional/country level	○	○	○	○
Other	○	○	○	○

Please comment:

20.2 ***L. major*** (human cutaneous leishmaniasis)

	0	1	2	3
Infected people and animals coming from endemic areas (travellers and migrants)	○	○	○	○
Sand fly vector expansion into previously-free areas as a result of climate change	○	○	○	○
Human-made environmental changes with an impact in vectors and reservoir hosts (e. g. urbanization, agricultural projects)	○	○	○	○
Insufficient/lack of surveillance at regional/country level	○	○	○	○
Insufficient/lack of prevention and control at regional/country level	○	○	○	○
Other	○	○	○	○

Please comment:

20.3 ***L. tropica*** (human cutaneous leishmaniasis)

	0	1	2	3
Infected people and animals coming from endemic areas (travellers and migrants)	○	○	○	○

Sand fly vector expansion into previously-free areas as a result of climate change				
Human-made environmental changes with an impact in vectors and reservoir hosts (e. g. urbanization, agricultural projects)				
Insufficient/lack of surveillance at regional/country level				
Insufficient/lack of prevention and control at regional/country level				
Other				

Please comment:

20.4 ***L. donovani*** (human visceral and cutaneous leishmaniasis)

	0	1	2	3
Infected people and animals coming from endemic areas (travellers and migrants)				
Sand fly vector expansion into previously-free areas as a result of climate change				
Human-made environmental changes with an impact in vectors and reservoir hosts (e. g. urbanization, agricultural projects)				
Insufficient/lack of surveillance at regional/country level				
Insufficient/lack of prevention and control at regional/country level				
Other				

Please comment:

H - CONCLUDING REMARKS

Please provide any additional information you consider relevant:
